# Elevated Level of *Fannyhessea vaginae* Correlated with Implantation Outcomes in Women undergoing *in vitro fertilization* Treatment

**DOI:** 10.4014/jmb.2606.06022

**Published:** 2026-10-13

**Authors:** Liya Paramita, Ching-Hao Teng, Mi-Chia Ma, Masayuki Hashimoto, Yen-An Tang, Hsien-An Pan, H. Sunny Sun

**Affiliations:** 1 Institute of Basic Medical Sciences, College of Medicine, National Cheng Kung University, Tainan, Taiwan; 2 Institute of Molecular Medicine, College of Medicine, National Cheng Kung University, Tainan, Taiwan; 3 Department of Statistics, National Cheng Kung University, Tainan, Taiwan; 4 An-An Women and Children Clinic, Tainan, Taiwan; 5 Center for Genomic Medicine, Research and Service Headquarter, National Cheng Kung University, Tainan, Taiwan; 6 Department of Obstetrics and Gynecology, College of Medicine, National Cheng Kung University, Tainan, Taiwan

**Keywords:** Embryonic loss, *Fannyhessea vaginae*, Implantation failure, Infertility, NGS, Vaginal microbiota

## Abstract

The vaginal microbiome plays a significant role in reproductive health. While maintaining a balanced vaginal microbiome is crucial for successful *in vitro fertilization* (IVF) outcomes, the specific bacteria associated with adverse outcomes may vary by region and are largely unknown. This study aims to investigate the bacterial profiles and their influence on embryo implantation, thereby supporting the development of microbiome-based therapies. One hundred women undergoing IVF provided vaginal swabs at three stages: oocyte retrieval, embryo transfer, and hCG pregnancy testing. An in-house 16S rRNA gene amplicon sequencing and bioinformatics pipeline was used to profile the vaginal microbiota. Bacterial communities were constructed, and the transitions across IVF stages were analyzed. We also used custom quantitative PCR assays to validate NGS results and provide species-level resolution for selected bacterial species. Unlike other regions where *Lactobacillus crispatus* predominates, we found that *Lactobacillus iners* is the main vaginal microbiota across all IVF stages in Taiwan. Nevertheless, significant shifts in bacterial community types were correlated with hormonal changes during IVF treatment. Importantly, while *Lactobacillus* levels didn't predict pregnancy success, higher levels of bacteria associated with bacterial vaginosis, particularly *Fannyhessea vaginae*, significantly correlated with implantation failure and early embryonic loss. The strong association between elevated *F. vaginae* levels and adverse implantation rate in Taiwanese women highlights the potential benefit of incorporating routine screening for this pathogen. Personalized microbiome interventions targeting *F. vaginae* may improve embryo implantation for women undergoing assisted reproductive technologies.

## Introduction

Infertility is a complex issue that significantly affects obstetric and perinatal outcomes [1, 2]. *In Vitro Fertilization* (IVF) has emerged as a successful solution, resulting in the birth of over five million babies worldwide [3-5]. However, many factors, including maternal dysfunction, embryonic chromosomal abnormalities, and maternal genital infections, can cause the failure of IVF treatment. In addition, several reproductive diseases, such as endometriosis [6], polycystic ovary syndrome (PCOS), adenomyosis, myoma [7], endometrial polyps (EPs) [8, 9], and ovarian cysts [10, 11], were correlated with diminished pregnancy rates. Altogether, it has resulted in an overall live birth rate for the first cycle of 29.5% (95% CI: 29.3, 29.7) in the UK [12], 32.7% (95% CI:32.2, 33.1%) in Australia and New Zealand [13], and 32.5% in the USA [14].

Among all genital tract infections, the vaginal microbiota plays a crucial role in reproductive health due to the disruption of the balance, leading to inflammation and chronic damage to the reproductive system, ultimately reducing fertility [15] and affecting various pregnancy outcomes from implantation to preterm birth [16]. A balanced *Lactobacillus*-dominated vaginal microbiota is associated with a healthy reproductive tract environment by producing lactic acid, which helps prevent infections and maintain immune balance [17]. Dysbiosis, or an imbalance in the microbiota, results in an overgrowth of pathogenic bacteria, leading to increased inflammation and immune disruptions [18]. The conditions potentially lead to an inhospitable uterine environment and increased risk of implantation failure or early pregnancy loss [11, 13], thus adversely affecting the early stages of pregnancy. This link highlights the importance of maintaining vaginal microbial balance and developing effective strategies to enhance IVF success.

Previous population studies have revealed that various factors, such as host genetics, diet, lifestyle, aging, and geographical location, are strongly associated with population-specific differences in the microbiome [19]. Regions with different diets [20], hygiene practices, sexual behaviors, and healthcare access may exhibit variations in the prevalence of vaginal pathogens [21]. These disparities affect the balance of *Lactobacillus* and the incidence of bacterial vaginosis (BV) and aerobic vaginitis (AV) [22]. Geographically distinct vaginal pathogens can increase inflammation and disrupt immune responses, thereby adversely affecting endometrial receptivity and embryo implantation. Researchers from diverse regions have investigated vaginal microbiomes in various cohorts, including those from the United States [23], China [24, 25], Europe [26], and other Asian populations [27]. These studies collectively demonstrate a connection between vaginal microbiota and IVF outcomes and suggest that an abnormal microbiome may negatively impact pregnancy outcomes. Understanding these geographic variations is crucial for developing targeted interventions and improving reproductive outcomes. Nonetheless, research specifically targeting geographically distinct pathogens impacting implantation remains limited.

Pathogens associated with BV and AV are prominent contributors to vaginal dysbiosis. BV is characterized by an imbalance in normal vaginal flora, typically exhibiting a low abundance of *Lactobacillus* species and an increase in anaerobic bacteria [28-31]. Although often asymptomatic, BV is linked to various reproductive health issues, including endometritis, pelvic inflammatory disease, and adverse pregnancy outcomes such as preterm delivery [32] and premature rupture of membranes (PROM) [33]. Conversely, AV is a polymicrobial vaginal infection characterized by an overgrowth of aerobic bacteria, such as group *B. Streptococcus*, *Escherichia coli*, and *Staphylococcus aureus*. While AV has often been overlooked in prior studies, leading to incomplete diagnostics and management [34], recent research has shown that AV is prevalent during late pregnancy and is associated with a range of negative pregnancy outcomes, including preterm birth and PROM [35]. Although the adverse effects of dysbiosis in BV and AV on pregnancy outcomes are recognized, the specific impact of these pathogens on early pregnancy after embryo transfer remains unclear.

Successful implantation is the critical first step for subsequent pregnancy outcomes. While numerous studies have focused on developing interventions to restore and maintain a healthy, *Lactobacillus*-dominant vaginal microbiome [36-38], research explicitly targeting the identification of geographically distinct pathogens that alter immune responses or produce harmful metabolites—thereby potentially diminishing the likelihood of successful implantation—remains limited. This study aimed to identify bacteria that negatively impact implantation, paving the way for microbiome-based therapeutics. Additionally, we analyzed microbiota profiles during the three phases of IVF treatment to determine the optimal timing for intervention. Using qPCR assays, we further investigated the association between specific bacterial species and IVF outcomes. Our findings confirm a significant association between the BV-related pathogen *F. vaginae* and implantation failure, supporting the need for a timely pre-treatment screening strategy to enhance implantation rates and ultimately contribute to the development of precision models for personalized microbiome therapeutics.

## Materials and Methods

### Participants and Sample Collection

Participants were recruited from the An An Women and Children Clinic in Tainan City, Taiwan. One hundred fifteen women of reproductive age were initially enrolled and provided informed consent; the Institutional Review Board (IRB) of National Cheng Kung University, Taiwan, approved the study (A-BR-107-003), and recruitment occurred from 2018 to 2019. Eligibility required that participants be 23–45 years old and comply with study protocols. Five participants dropped out during the study, including two participants who experienced massive bleeding. An additional 10 participants did not have available pregnancy outcomes. Therefore, we used microbiota profiles from 100 participants with available pregnancy outcomes for final data analysis. A formal power calculation was not performed *a priori* due to the pilot and exploratory nature of this study. The sample size was chosen based on feasibility and resource constraints.

Vaginal swabs were obtained at the three phases of IVF treatment—oocyte retrieval, embryo transfer, and hCG pregnancy confirmation —by trained professionals using Sterile Libo Specimen Collection and Transport Swabs (Libo Medical Products Inc., Taiwan) from the mid-vaginal wall (Supplementary Fig. S1). Collected swabs were placed in microcentrifuge tubes, stored at -20°C, and then transported on a cooler to the Institute of Molecular Medicine at National Cheng Kung University in Tainan City.

### Ovarian Stimulation, Oocyte retrieval, and Embryo Culture

Participants underwent IVF cycles and stimulation protocols in accordance with institutional guidelines. Ovarian stimulation utilized an antagonist protocol involving recombinant FSH (r-FSH) (Gonal-F; Merck Serono, Germany) and human menopausal gonadotropins (hMG) (Menopur^®^; Ferring Pharmaceuticals, Switzerland). On day 3 of the menstrual cycle, participants received 225-300 IU of rFSH, with doses adjusted based on ovarian response, as assessed by transvaginal ultrasonography and serum hormone levels. GnRH-A (0.25 mg, Cetrorelix; Merck Serono, Germany) was administered daily once the leading follicles reached a diameter of 14 mm. When more than two follicles reached 17 mm, or the leading follicles reached 18 mm, r-hCG (Ovidrel; Merck Serono, Germany) was given subcutaneously.

Oocyte retrieval was conducted 36 hours post-r-hCG injection using transvaginal ultrasound-guided double-lumen needle aspiration. The retrieved oocytes were inseminated through conventional IVF or intracytoplasmic sperm injection (ICSI) 4 to 6 hours after retrieval. Cumulus cells were removed entirely, and fertilized oocytes were cultured in an embryo culture medium (Global^®^ Total^®^; CooperSurgical, CT, USA) under controlled conditions of 37°C, 5.5% CO2, and 5% O2 until further procedures were indicated. Only frozen-thawed embryo transfer (FET) was employed in this study.

### Pregnancy Outcomes

The primary outcome was determined by a positive pregnancy test, indicated by serum β-hCG levels of 20 IU/L or higher, measured 12 days after embryo transfer. The secondary outcome is clinical pregnancy, defined by the ultrasonographic visualization of one or more gestational sacs. Chemical pregnancy refers to a very early miscarriage where an initial positive indication of pregnancy is detected by serum β-hCG. Still, the embryo stops developing, and no gestational sac is visible on ultrasound.

### DNA Extraction and Amplicon Library Preparation

The vaginal swabs were initially stored at -20°C and thawed on ice before extraction. Genomic DNA was extracted from vaginal swab specimens using the DNeasy PowerSoil Kit (Qiagen, Germany) with the Minilys homogenizer (Bertin Technology, Montigny-le-Bretonneux, France). DNA isolation was performed according to the manufacturer’s protocol, resulting in genomic DNA eluted in 50 μL of elution buffer, which was stored at -20°C for subsequent analysis.

To amplify the V3–V5 region of the 16S rRNA gene, a custom polymerase chain reaction (PCR) protocol was utilized to produce an amplicon of approximately 560 bp (V3-357F: 5ʹ-CCTACGGGAGGCAGCAG-3ʹ and V5-926R: 5ʹ-CCGTCAATTCMTTTRAGT-3ʹ). Barcoded libraries were quantified using the StepOnePlus Real-time PCR System (Thermo Fisher Scientific, USA) with the KAPA Library Quantification Kit (Roche, Switzerland).

### Sequencing and Data Analysis

The DNA library was diluted to a final concentration of 12.5 pM, mixed with a 20% PhiX control, and loaded onto a next-generation sequencing (NGS) platform. Paired-end sequencing generated 300 bp reads in both the forward and reverse directions using the MiSeq Illumina Sequencer (Illumina, USA). Each NGS run included pooling 40 to 90 sample libraries, targeting a final mapped high-quality read count of more than 150,000 reads per sample.

The Binary Base Call (BCL) files were converted into FASTQ files and analyzed using the QIIME 1.9 pipeline. Sequence quality was improved through trimming with Trimmomatic version 0.38, with a minimum sequence length of 100 bp. Forward and reverse sequences were merged using FLASH 1.2.11, with minimum and maximum overlapping sequences set to 10 bp and 65 bp, respectively. Sequences were filtered for length, end-trimming, and a minimum Phred quality score of 0.001 to ensure high data quality. Chimeric sequences resulting from PCR amplification of multiple templates were removed, yielding purified abundance sequences using USEARCH v6. Operational taxonomic units (OTUs) were generated using the UCLUST program in QIIME, applying a 97% sequence similarity threshold for taxonomic classification. The original sequences were cross-referenced against the Ribosomal Database Project (RDP) database and subsequently reassigned to the Greengenes database for reference. Additionally, alpha diversity was assessed, and rarefaction curves were generated. The Shannon index was used to assess biodiversity across the sample collection and to generate weighted and unweighted beta diversity metrics.

### Vagitype Assignment and Ternary Plot Analysis

The vagitype assignment was conducted using a previously established methodology based on 16S taxonomic profiles [39]. Briefly, samples were categorized into vagitypes by identifying the taxon with the highest percentage of reads in each sample, applying a minimum representation threshold of 30%. Samples that did not meet this threshold and lacked a dominant taxon were classified as “no type.”

A ternary plot was generated to visualize relationships among the selected OTUs across the three IVF stages (VS1, VS2, and VS3). The analysis focused on the top 20 most abundant OTUs at the species level, using overall abundance values from patients with the identified OTUs. Patients lacking a specific OTU were excluded from further calculations. Each data point on the ternary plot represents a single OTU, with coordinates determined by the average relative abundance of that OTU at each stage, normalized to 1. The size of each circular data point was scaled to reflect the frequency of that particular OTU within the studied population.

### Analysis of Species Abundance and Taxa Enrichment

We followed established procedures from prior research to analyze species abundance and identify significantly enriched taxa within each group [39]. Initial filtering removed low-abundance taxa based on two criteria: taxa were retained if present in at least 15% of profiles with an abundance of at least 0.001 (0.1%) or found in at least 5% of profiles with an abundance of at least 0.01 (1%). Taxa that did not meet these criteria were excluded from further analysis, and abundance values below 0.0001 (0.01%) were rounded to zero for the remaining taxa.

### Measurement of Bacterial Loads Using TaqMan Assays

To quantify bacterial loads in each sample, we developed TaqMan assays (TaqMan^®^ Assay for Vaginal Microbiota Profiling, Life Technologies Co., USA) targeting two bacterial vaginosis pathogens, *F. vaginae* and *P. bivia*, as well as two *Lactobacillus* species, *L. iners* and *L. crispatus*. TaqMan assays targeting prokaryotic 16S rRNA served as a reference for the total bacterial load in each individual. Pooled DNA from all participants was utilized as an internal control for cross-sample comparisons.

TaqMan assays were conducted using specific probes on the StepOnePlus Real-Time PCR System (Thermo Fisher Scientific, USA). qPCR data were analyzed using StepOnePlus 2.3 software (Applied Biosystems, USA). Each amplicon was run in triplicate in separate wells to quantify target bacteria relative to the prokaryotic 16S rRNA gene (reference control). The threshold cycle number (Ct) was averaged from the triplicate PCR reactions, with consistent threshold and baseline settings across all samples. The Ct value for each assay indicated bacterial abundance in an individual, and the relative abundance of target bacteria was determined using the ΔCt method, which involved subtracting the Ct value of the target bacteria from that of total bacteria.

The proportion of target bacteria to total bacteria was calculated as 2^ΔCt^. To compare target bacterial loads across samples, an internal control (pooled DNA from all participants) was used, and the ΔΔCt value was calculated by subtracting the ΔCt value from the control sample. The corresponding relative abundance of the target bacteria was then calculated as 2^ΔΔCt^.

### Statistical Analysis

Statistical analyses were performed using GraphPad Prism version 8 (GraphPad Software, Inc., USA). Differences in categorical variables were evaluated using the Chi-squared test. Continuous variables were expressed as mean ± standard deviation (SD) or median (interquartile range, IQR), depending on the data distribution. Comparisons between two independent groups were performed using the non-parametric Mann–Whitney U test or the unpaired t-test, while repeated measurements across IVF stages were analyzed using the Friedman test. Statistical significance was defined as *p* < 0.05.

Microbiome sequence data were processed and analyzed using QIIME version 1.9.1. Alpha diversity was assessed using the Shannon diversity index and compared between groups using the Mann–Whitney U test or Friedman test, as appropriate. Beta diversity was evaluated using weighted and unweighted UniFrac distance metrics and visualized by principal coordinate analysis (PCoA). Relative abundances of bacterial genera and taxa between the chemical and clinical pregnancy outcome groups were compared using the unpaired t-test after excluding samples with zero relative abundance for the corresponding taxa.

Data visualization, including stacked bar charts and alluvial plots, was generated using the ggplot2 package in R version 4.0.5 (R Foundation for Statistical Computing, Austria). Ternary plots of the top 20 operational taxonomic units (OTUs) were generated using Grapher version 17 (Golden Software, USA).

For qPCR analyses, we quantified bacterial abundance using the ΔCt method and compared groups using the Mann–Whitney U test. We evaluated agreement between qPCR and NGS measurements using simple linear regression and reported the strength of association as Pearson's correlation coefficient (r) and the coefficient of determination (R^2^).

## Results

### Demographic and Clinical Characteristics of the Study Population

The clinical and demographic characteristics of the study population are summarized in Table 1. Among the 115 women of reproductive age recruited, the average age was 35 years (range: 23-45 years). Fifteen participants were excluded due to incomplete sampling, resulting in a final cohort of 100 participants. Within this group, 59 women were categorized as of advanced maternal age. Based on human chorionic gonadotropin (hCG) tests, participants were divided into two groups: hCG-positive (hCG+, N = 70) and hCG-negative (hCG-, N = 30). Clinical pregnancy using ultrasound assessments identified 53 participants as pregnant (P) and 47 as non-pregnant (NP).

A marginal age effect was observed (P = 0.056) when comparing pregnancy outcomes by hCG results across age groups (Supplementary Table S1). The difference reaches significance and reveals higher pregnancy rates among participants younger than 35 years (*P* < 0.05) concerning clinical pregnancy status (Table 1). Other factors, including reproductive disease, surgical history, primary vs. secondary infertility, previous infertility treatments, menstrual cycle conditions, and hormonal therapy, did not demonstrate statistical differences in pregnancy outcomes (Table 1). Notably, participants without a prior history of vaginal infection showed a significant association with higher pregnancy rates (*p* < 0.05), highlighting the important influence of vaginal infection in achieving successful pregnancies.

### Unique Vaginal Microbiota Profiles across Various IVF Treatments

Three hundred samples from 100 women were collected at three key time points: egg retrieval (VS1, N = 100), embryo transfer (VS2, N = 100), and the hCG pregnancy test (VS3, N = 100) to profile the vaginal microbiota dynamics throughout different stages of IVF treatment. DNA was isolated from these samples and subjected to 16S rRNA gene amplicon sequencing, followed by data analysis (Supplementary Fig. S1). The V3-V5 regions of the 16S rRNA gene were successfully amplified and sequenced using an in-house developed pipeline. In total, 56,388,353 reads from all 300 samples were analyzed. The number of amplicon sequences assigned to each sample ranged from 50,834 to 588,868, with a mean of 187,961 sequences per sample. Core analysis identified 452 unique OTUs at the genus level and 610 at the species level.

Microbiota diversity of the vaginal samples was assessed using the Shannon index and distinct vaginal microbiota compositions were observed at these time points (*p* < 0.05, Fig. 1A). Specifically, a lower diversity was recorded at the VS3 stage (1.66) compared to the VS1 stage (1.99, *P* < 0.05) and the VS2 stage (1.92, *P* = 0.2), indicating a reduction in microbiota diversity at the time of pregnancy establishment (*i.e.*, hCG pregnancy test). No differences in beta diversity were detected among the three stages, as indicated by UniFrac analyses, which showed both weighted (*P* = 0.08, Fig. 1B, left) and unweighted (*P* = 0.29, Fig. 1B, right) metrics, suggesting a comparable overall number of bacterial species in samples across the stages. Additionally, there were no differences in the relative abundance of bacterial profiles among the treatment stages. These observations suggest that IVF treatments distinctly influence species abundance and richness within individual participants without altering the presence or absence of specific taxa, thereby maintaining similar community compositions across IVF stages.

### Vaginal Microbiota Composition and Diversity throughout IVF Treatment

To investigate microbiota characteristics across the three stages of IVF treatment, we analyzed OTUs from all 300 profiles within each stage group. At the species level, the distribution summary of the top 20 bacterial taxa across the three stages (Fig. 1C and Supplementary Table S2) revealed that *L. iners* (35%−45.9%) and *Lactobacillus* spp. (26.1%−42.7%) were the dominant groups, collectively accounting for over 70% of the identified taxa. Additional taxa that exhibited a prevalence of at least 1% across all stages included *F. vaginae* (4%−7.4%), *Streptococcus agalactiae* (2.5%-6.2%), *Prevotella* spp. (1%−5.8%), *Streptococcus anginosus* (1%−1.9%) and *Gardnerella* spp. (1%−2.4%).

Eleven out of 20 OTUs were detected in more than 80% of patients, suggesting that these bacteria may be essential constituents of the reproductive tract microbiome. In addition, seven OTUs are present in less than 1% of the population (Supplementary Table S2), indicating variability in specific microbial populations across stages. We further generated a ternary plot (Fig. 1D) to analyze the differential species based on the top 20 species. While many OTUs, including *L. iners* and *Lactobacillus* spp., were positioned centrally, indicating similar abundance and composition across the three stages, *Sneathia* spp. and *Lactococcus garvieae* or *Lactococcus* spp. were located at the edges of VS1 and VS3, suggesting that these taxa exhibited unique profiles at stages 1 and 3. These findings highlight a stable, *Lactobacillus*-dominated microbiome across IVF stages, with certain species exhibiting distinct distribution patterns at specific stages of IVF treatment.

### Temporal Changes in Vaginal Microbial Community Structure across IVF Stages

Next, we applied the vagitype method to assess the taxonomic composition of the samples and construct microbial community profiles. Except for two samples that presented a profile classified as no-vagitype, a total of 16 distinct vaginal communities were identified from the 298 profiles analyzed (Table 2). The vagitype assignments indicated that nearly all samples were predominantly composed of *Lactobacillus* species (Fig. 2A), with the most prevalent species being *L. iners* in VS1 (47%) and VS2 (49%) and *Lactobacillus* spp. in VS3 (43%). Furthermore, an increase in *Lactobacillus*-dominated vagitype was observed, rising from 71% in VS1 to 79% in VS2 and reaching 80% in VS3. Among the groups, nine vagitypes were present in individuals across at least two stages, while eight vagitypes were identified in only 1-3 individuals within a single stage. Notably, six new vagitypes emerged exclusively at stage 3, with each identified in fewer than three individuals. These findings collectively illustrate the dynamic nature of the vaginal microenvironment, which undergoes distinct changes throughout each stage of IVF treatment.

To investigate community changes in vaginal microbiota across three IVF stages, we grouped the 17 identified vagitypes into five clusters: *Lactobacillus*-related (including *L. iners*, *Lactobacillus* spp., and *Lactobacillus* reuteri), BV-associated bacterium (BVAB)-related (including *F. vaginae*, *Prevotella* spp., *Gardnerella* spp., and *Sneathia* spp.), AV-associated bacterium (AVAB)-related (including *S. agalactiae*, *Streptococcus anginosus*, *Streptococcus luteciae*, *E. coli*, and *Klebsiella* spp.), *Lactococcus*-related (including *Lactococcus* spp. and *Lactococcus garvieae*), and others. Although *L. iners* may have distinct ecological and clinical characteristics from other *Lactobacillus*-related species, it was included within the *Lactobacillus*-related cluster to characterize community-level transitions rather than species-specific clinical associations. While *Lactobacillus*-related vagitypes showed dominance, significant shifts were observed in the abundance of these groups across the IVF stages (Fig. 2B). A trend from BVAB- and AVAB-related vagitypes towards a predominance of *Lactobacillus*-related vagitypes suggests a more stable vaginal environment.

Notably, *Lactobacillus*-related vagitypes exhibited remarkable stability (Fig. 2C), with 96% of participants maintaining their profiles from VS1 to VS2 and 94% from VS2 to VS3. In contrast, BVAB-related participants decreased from 18 in VS1 to 5 in VS3, while some transitioned to *Lactobacillus*-related vagitypes. Approximately 10% of participants in each stage were classified as AVAB, but these individuals differed between stages, representing less stability within this group (Fig. 2C). Interestingly, some individuals shifted from *Lactobacillus* to AVAB-related vagitypes in VS3, with *Klebsiella* noted as a prominent bacterium (Table 2). These findings underscore the dynamic changes in the vaginal microbiome during IVF that may impact treatment success.

### Significant Association of BVAB and Implantation Failure

Our analysis focused on hCG pregnancy test results as an indication of pregnancy establishment. Comparisons of microbiota diversity revealed no significant differences between hCG-positive and hCG-negative women throughout the IVF treatment stages (Fig. 3A). The Shannon index showed the lowest diversity at the VS3 stage; however, no differences were observed between the groups (P = 0.63). Similarly, beta diversity analyses yielded no significant differences (Fig. 3B). The reduced diversity at VS3 likely reflects a uniform rise in *Lactobacillus* dominance; however, the lack of differentiation by pregnancy outcomes suggests that *Lactobacillus* alone does not dictate pregnancy success.

We analyzed bacterial composition alongside pregnancy outcomes from hCG tests to identify key bacteria influencing early pregnancy. Microbiota profiles were cleaned by filtering out the low-abundance taxa and rounding sample abundance values below 0.0001 (0.01%) to zero, resulting in nine genera and 14 species for analysis. While no genera were found to enhance pregnancy outcomes, two BVAB-related bacteria—*Fannyhessea* (0.089 ± 0.045 vs. 0.019 ± 0.010, *p* < 0.05) and *Prevotella* (0.024 ± 0.019 vs. 0.002 ± 0.001, *p* < 0.05)—showed significantly higher proportions in hCG-negative groups (Fig. 3C). These results suggest that BVAB negatively impact embryo implantation potential and contribute to pregnancy failure, potentially through interactions with the local inflammatory microenvironment.

### Association of BVAB-Related Bacteria with Implantation Failure and Early Embryonic Loss

Chemical pregnancy refers to an initial positive pregnancy test result followed by a negative detection at the clinical pregnancy examination, indicating an early embryonic loss. To investigate specific bacteria associated with implantation failure and early embryonic loss, we analyzed vagitype profiles at VS3 (the time of the hCG test) and assessed their associations with pregnancy outcomes at hCG measurement and clinical assessment. Out of the 100 women in the study, 70 tested positive for pregnancy at the two-week mark following embryo transfer (Fig. 4A, VS3 to hCG status). Of note, all five BVAB vagitype patients tested as hCG-negative, indicating implantation failure. Furthermore, the number of hCG+ patients decreased to 53 after confirmed clinical pregnancies were identified by ultrasound at approximately 6 weeks, resulting in a high chemical pregnancy rate of 24.3% (17/70; Fig. 4A, hCG+ to clinical pregnancy). We then tracked vagitype transitions among the 17 women who experienced early embryonic loss (Fig. 4A, hCG+ to no clinical pregnancy). Of these, 13 were classified as having *Lactobacillus*-dominant vagitypes at the VS3 stage, while four belonged to the AVAB group, dominated by *Klebsiella* (in two cases, 45% and 82%), *Enterococcaceae* (one case, 82%), and *E. coli* (one case, 66%). We next analyzed microbiota profiles associated with clinical pregnancy outcomes to explore whether specific bacteria may influence early embryonic loss and contribute to a chemical pregnancy. Interestingly, while no association was detected in the population at the time of pregnancy test, we found that the proportion of *Klebsiella* in the non-pregnant (NP) group was significantly higher than that in the pregnant (P) group (0.09 ± 0.038 vs. 0.009 ± 0.005, *p* < 0.05, Fig. 4B). The results support the potential negative influence of AVAB bacteria, especially *Klebsiella*, on early pregnancy loss.

Our data demonstrated that patients with *Lactobacillus*-dominant vagitypes can also result in early pregnancy failure, indicating that *Lactobacillus* dominance at the community level alone may not be sufficient to predict pregnancy success, potentially due to other contributing factors such as embryonic chromosomal abnormalities. Nevertheless, all five patients with BVAB-dominant vagitypes, who showed high levels of BVAB pathogens exceeding 45% in this study, failed to establish a pregnancy (Fig. 4C). Collectively, this indicates that elevated BVAB pathogens significantly contribute to early embryonic stage pregnancy failure.

### Validation of NGS Vaginal Microbiota Profiling Using qPCR

While NGS is widely used for comprehensive vaginal microbiota profiling, its high cost and time demands limit routine clinical application. In addition, although 16S rRNA gene sequencing provides an overview of the microbial community, its taxonomic resolution may be insufficient for reliable species-level discrimination of closely related taxa. To address these limitations, we developed quantitative polymerase chain reaction (qPCR) assays to validate NGS findings and provide relatively low-cost assays with fast run times and species-specific resolution. We established and applied TaqMan assays to detect specific bacteria in 300 DNA samples. Two BVAB pathogens, *F. vaginae* and *Prevotella* spp., and one *Lactobacillus* spp., *L. iners*, were detected in the NGS profiles; thus, the proportions of these specific bacteria obtained by qPCR were compared with the corresponding NGS data for each individual to provide a robust framework for confirming NGS-derived microbiota profiles. We found significant and high positive correlations for *F. vaginae* (r = 0.91, P < 0.001, Fig. 5A, upper left), *P. bivia* (r = 0.78, *p* < 0.001, Fig. 5A, upper right), *L. iners* (r = 0.53, P < 0.001, Fig. 5A, lower left), and *L. crispatus* (r = 0.46, *p* < 0.001, Fig. 5A, lower right) between the two datasets, indicating effective replication of NGS results by qPCR. However, 242, 229, 121, and 188 samples were undetectable in qPCR assays for *F. vaginae*, *P. bivia*, *L. iners*, and *L. crispatus*, respectively, with average undetected percentages of 0.19%, 0.35%, 7.99%, and 16.46%. This suggests that the 16S rRNA-based NGS technology used in our study provides a broader view of microbial diversity and better detection of low-abundance species.

In 300 samples, *Lactobacillus* was detected at the genus level. Among the identified *Lactobacillus*-dominant vagitypes, *L. iners* was the leading species, representing 57.6% (133/231), and the remaining cases were dominated by other *Lactobacillus* spp. that could not be differentiated from our NGS pipeline. We applied a qPCR assay to examine *L. crispatus*, the commonly dominant species among *Lactobacillus* spp. We found a 34.3% detection rate for *L. crispatus*, with 23.3% of cases showing a detection rate below 1%. Both *L. iners* and *L. crispatus* were detected in 51 samples, while 68 samples (22.7%) lacked these species, underscoring the diversity of *Lactobacillus* spp. among Taiwanese women (Fig. 5B). Overall, these findings support the utility of qPCR in highlighting vaginal *Lactobacillus* species diversity and suggest that *Lactobacillus* spp., other than *L. iners* and *L. crispatus*, are commonly present in the vagina in Taiwanese women undergoing IVF treatment.

### *Fannyhessea vaginae* Abundance as a Predictive Marker for IVF Pregnancy Success in Taiwanese Women

We then studied the associations between specific bacteria and pregnancy outcomes. The analysis revealed similar relative abundances of *L. iners* and *L. crispatus* between hCG-positive and hCG-negative patients, aligned with NGS findings (Fig. 5C). In contrast, non-pregnant patients had significantly higher levels of *F. vaginae* (*p* < 0.05), while *P. bivia* levels showed no association with hCG-negative outcomes (*p* = 0.35). Notably, successful clinical pregnancies were associated with significantly higher levels of *L. iners* (*p* < 0.01), whereas *F. vaginae* exhibited higher levels in non-pregnant patients (*p* < 0.05) at the clinical pregnancy stage (Fig. 5D). These findings suggest that *L. iners* may serve as a positive bacterial marker for pregnancy, while *F. vaginae* may indicate pregnancy failure in IVF patients, especially among Taiwanese women.

Although the role of *L. iners* in enhancing pregnancy showed inconsistencies across NGS and qPCR assays, *F. vaginae* consistently correlated with early pregnancy outcomes. To assess the clinical utility of these findings, we examined *F. vaginae* levels across the three IVF stages to determine the time point at which its abundance showed the strongest association with successful implantation. Among 56 *F. vaginae*–positive samples (31 from pregnant patients, 25 from non-pregnant patients), levels varied among individuals and across IVF stages, but overall tended to decline toward the final sampling point (VS3) in patients who achieved pregnancy. Taken together, these results indicate that lower *F. vaginae* levels at the time of embryo transfer are associated with successful implantation, whereas higher levels may be associated with failure. Further study is warranted to confirm these findings and to define precise thresholds for clinical use.

## Discussion

The vaginal microbiome's formation and evolution begin at birth, shaped by genetic, dietary, lifestyle, and environmental factors [40, 41]. Studies such as the Vaginal Human Microbiome Project (VaHMP) and the Multi-Omics Microbiome Study (MOMS) highlight variations in vaginal microbiome profiles across different ethnic groups, particularly during pregnancy. Our research focuses on identifying bacterial community types that negatively influence implantation, thereby supporting the development of microbiome-based therapies. We observe that, despite *Lactobacillus* vagitypes being predominant, distinct vaginal microbiota profiles vary across different phases of IVF treatment. Notably, the significant shifts in microbiota prevalence illustrate the dynamic nature of the microbiome during IVF.

Hormonal fluctuations, particularly in estrogen and progesterone levels, affect the vaginal microbiota by altering vaginal pH, nutrient availability, and immune responses, thereby influencing overall vaginal health and susceptibility to conditions [42]. Controlled ovarian stimulation (COS) combined with progesterone luteal supplementation before IVF induces the development of multiple ovarian follicles [43] and alters vaginal and endometrial microbiota, increasing the presence of potentially pathogenic genera [44]. Our findings indicate that alpha diversity peaks at the egg retrieval stage. In addition, increases and decreases in the proportions of *Lactobacillus*-dominant and AVAB/BVAB-dominant vagitypes, respectively, from VS1 to VS2 were observed, indicating that the microbiota profile at the time of oocyte retrieval is significantly affected by hormonal treatment and suggesting that interventions may be most effective once a stable microbiota profile is established.

The composition of the vaginal microbiota plays a critical role in reproductive health, with microbial diversity linked to pregnancy outcomes. Previous research has linked microbial diversity to pregnancy outcomes, emphasizing the role of *Lactobacillus* spp. in sustaining low diversity, maintaining the functional balance in the vaginal environment, and promoting successful embryo attachment [45, 46]. With species-specific probes, we found that 59.7%, 34.3%, and 22.7% of samples detected *L. iners*, *L. crispatus*, *and L.* others, respectively. While *L. iners* and *L. crispatus* are commonly dominant in many populations, our study identified distinct patterns among Taiwanese women, suggesting geographic differences in predominant *Lactobacillus* species. The observed variability in vaginal microbiota composition—potentially influenced by genetic, environmental, or lifestyle factors—underscores the need to consider regional differences in microbiota profiling.

We identified 17 distinct vagitypes, primarily characterized by *Lactobacillus* species and *L. iners*, followed by *F. vaginae*, *S. agalactiae*, and *Prevotella* spp. Our findings align with previous studies that identified five community state types (CSTs) in vaginal microbiota [47, 48]. The *L. iners*-dominant vagitype corresponds to CST III, while other *Lactobacillus* vagitypes are associated with CST I, II, and V. The remaining vagitypes fall under CST IV, characterized by non-*Lactobacillus* dominance. Notably, patients with *Lactobacillus*-dominant vagitypes showed greater stability in microbial composition than those with BVAB or AVAB vagitypes, who experienced more shifts throughout IVF stages. This variability highlights the utility of vagitypes for understanding microbiota variation and its potential associations with reproductive outcomes. As the current study was conducted on women undergoing IVF without preimplantation genetic testing for aneuploidy, the observed pregnancy failure cannot rule out the influence of chromosomal abnormalities in transferred embryos. Thus, although 80% of participants had a *Lactobacillus*-dominant vagitype, we did not find a direct association between *Lactobacillus* species and pregnancy outcomes—potentially due to the ploidy of transferred embryos. In contrast, species-specific qPCR analysis showed that higher *L. iners* abundance was significantly associated with successful clinical pregnancy outcomes in our study population. Taken together, these findings suggest that overall *Lactobacillus*-dominant community type alone may not be sufficient to predict implantation or pregnancy success, *Prevotella*, were significantly specific *Lactobacillus* species may have distinct associations with reproductive outcomes.

Nevertheless, we found specific BVAB taxa, particularly *F. vaginae* and *P. bivia*, were significantly associated with implantation failure. Previous studies have shown that BVAB in the vaginal microbiome is linked to molecular alterations that activate toll-like receptor signaling, leading to localized inflammatory responses [49] and triggering pro-inflammatory cytokine production [50]. These findings support the notion that elevated levels of BVAB alter the vaginal microenvironment, potentially compromising endometrial receptivity and the immune-tolerance processes essential for successful embryo implantation, thereby resulting in substantially reduced implantation rates.

We successfully developed qPCR assays that correlate well with NGS data, making them a feasible option for routine clinical use due to their cost-effectiveness and faster processing times [51]. In addition, the detection of a strong association between the level of *F. vaginae* and implantation failure in IVF patients highlights the significance of microbial screening and management as part of IVF treatment to improve successful pregnancy rates. Importantly, incorporating routine screening for *F. vaginae* into clinical screening to identify patients at high risk of pregnancy failure before and during IVF treatment will enable personalized interventions, such as tailored antibiotic or probiotic treatments based on individual microbiota profiles, thereby potentially enhancing IVF success rates. However, large-scale clinical trials are essential to validate the safety, efficacy, and cost-effectiveness of these approaches, with long-term follow-up needed to evaluate their impact on pregnancy outcomes and vaginal health.

In conclusion, a balanced vaginal microbiome is important for successful IVF outcomes for women worldwide, though the specific bacterial species associated with adverse outcomes may vary by region. Using both NGS and qPCR, we identified that *F. vaginae*, a bacterial vaginosis-associated bacterium, strongly correlated with implantation failure and early pregnancy loss in Taiwanese IVF patients. Understanding specific bacterial species and their prevalence provides valuable insights for personalized microbiome interventions aimed at maintaining a *Lactobacillus*-dominant vaginal microbiome while minimizing BVABs. Finally, we underscore the necessity for routine screening and management of the reproductive microbiome to optimize pregnancy outcomes.

## Supplemental Materials

Supplementary data for this paper are available on-line only at http://jmb.or.kr.



## Figures and Tables

**Fig. 1:**
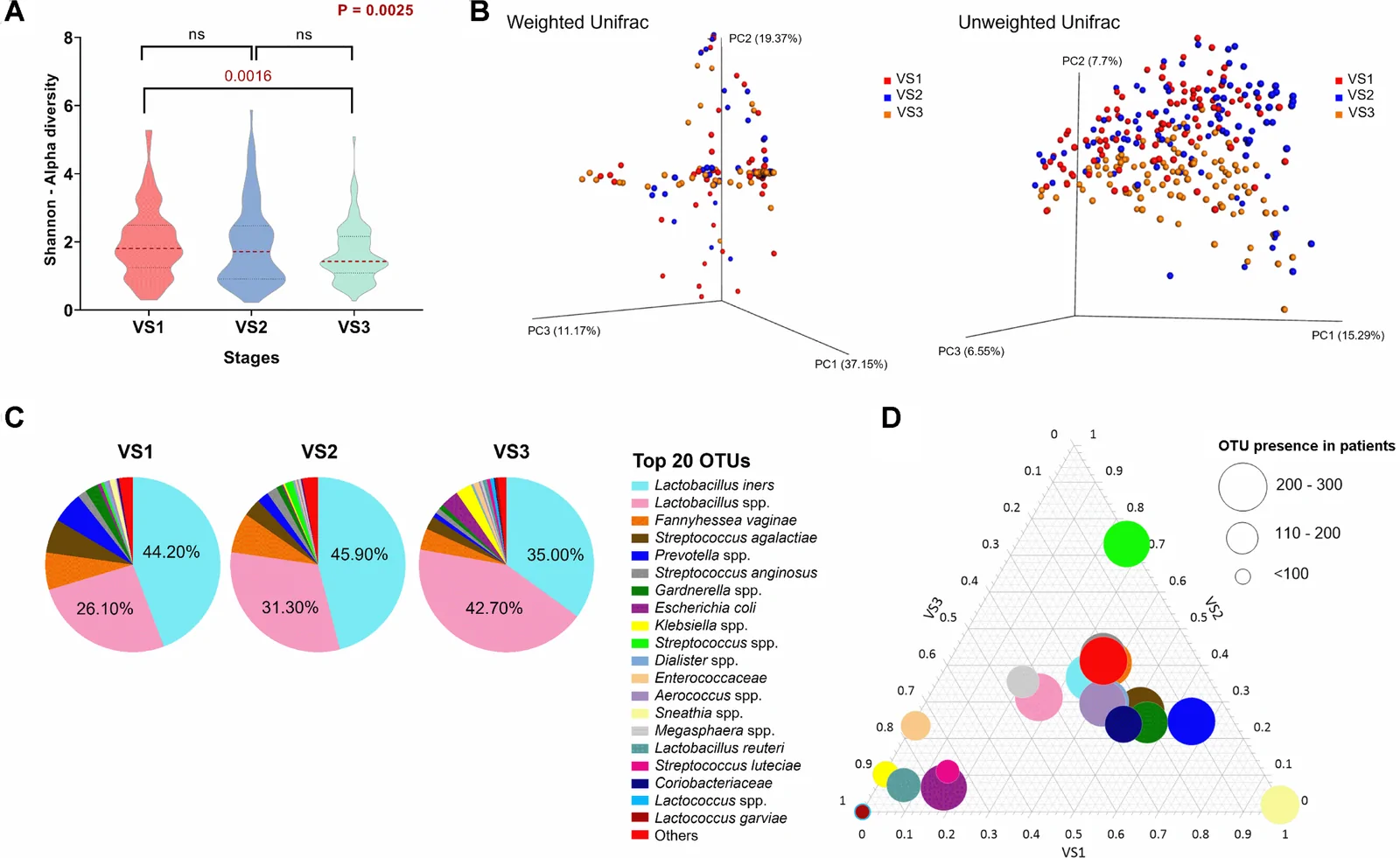
Microbiota composition and diversity across three stages during egg retrieval (VS1), embryo transfer (VS2), and the hCG pregnancy test (VS3). (**A**) Violin plot comparing the Shannon diversity index across the three stages (VS1, VS2, VS3). Higher values indicate greater diversity. (**B**) 3D plots visualize beta diversity using weighted and unweighted UniFrac distance metrics. Each point represents a sample, colored by disease stage. (**C**) Three pie charts showing the relative abundance of different bacterial taxa within each stage (VS1, VS2, VS3). Percentages indicate the proportion of each taxon. (**D**) A ternary plot illustrating dominant bacterial taxa's relative abundance and distributions across samples. Each data point on the ternary plot represents a single OTU, with coordinates determined by the average relative abundance of the OTU in each stage, normalized to 1. The size of each circular data point was scaled to reflect the frequency of that particular OTU within the sample. The color key indicates the bacterial species represented. The plot helps visualize the overall proportions of these species. The statistical significance levels: * *P*-value < 0.05, ** *P*-value < 0.01, *** *P*-value < 0.001.

**Fig. 2:**
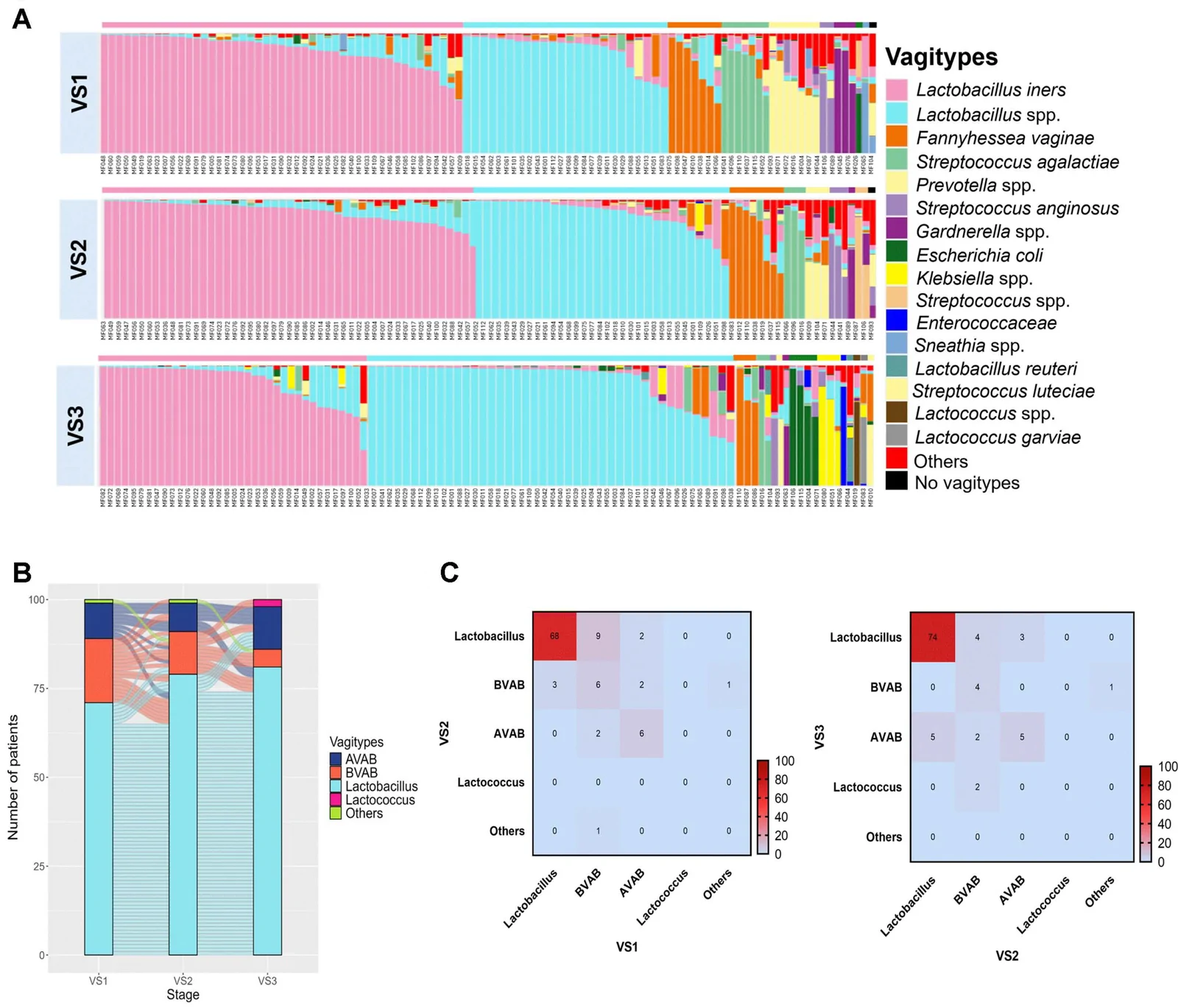
Vaginal Microbiota Community Types and Transitions Across Three Stages of IVF. (**A**) Stacked bar chart showing the relative abundance of various bacterial genera within each vaginal sample across the three IVF stages (VS1, VS2, VS3). Each bar represents a sample, and the segments within the bar indicate the proportion of each bacterial genus. A legend identifies the different vagitype (dominant genera) in each sample. (**B**) An alluvial plot illustrating the overall relative abundance and changes in the flow of the major bacterial community states (vagitypes) across the three IVF stages. (**C**) Two heatmaps display the number of transitions between different vagitypes. Left Heatmap (VS1-VS2) shows the number of transitions from vagitypes in VS1 to vagitypes in VS2. The right Heatmap (VS2-VS3) shows the number of transitions from vagitypes in VS2 to vagitypes in VS3. The color scale represents the transition counts. Darker red indicates more transitions between the specific vagitype pairs.

**Fig. 3:**
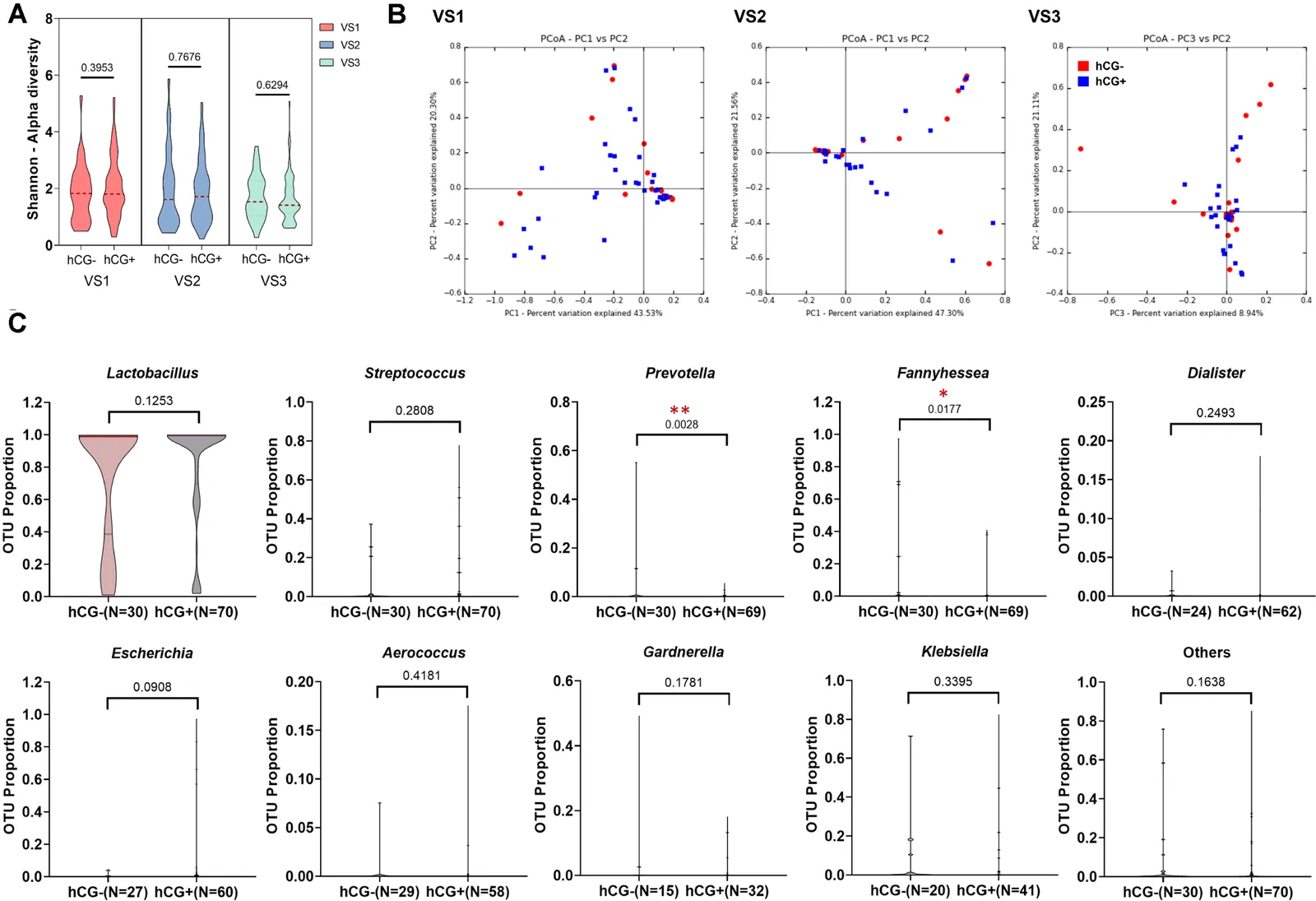
Association of Vaginal Microbiota with hCG pregnancy test Outcomes. (**A**) Violin plot comparing the Shannon diversity index of the vaginal microbiota across different IVF stages (VS1, VS2, VS3) for both hCG-positive and hCG-negative groups. (**B**) The 2D PCoA plots showing beta diversity of the vaginal microbiota in VS1 (left), VS2 (middle), and VS3 (right), distinguishing hCG-positive (blue dots) and hCG-negative (red dots) groups. Each point represents a sample. (**C**) Violin plots displaying the association of relative abundance of several key bacterial genera at the Genus level (*Lactobacillus*, *Streptococcus*, *Prevotella*, *Fannyhessea*, *Dialister*, *Escherichia*, *Aerococcus*, *Gardnerella*, *Klebsiella*, and Others) and hCG-positive and hCG-negative outcomes. The statistical significance levels: * *P*-value < 0.05, ** *P*-value < 0.01, *** *P*-value < 0.001.

**Fig. 4:**
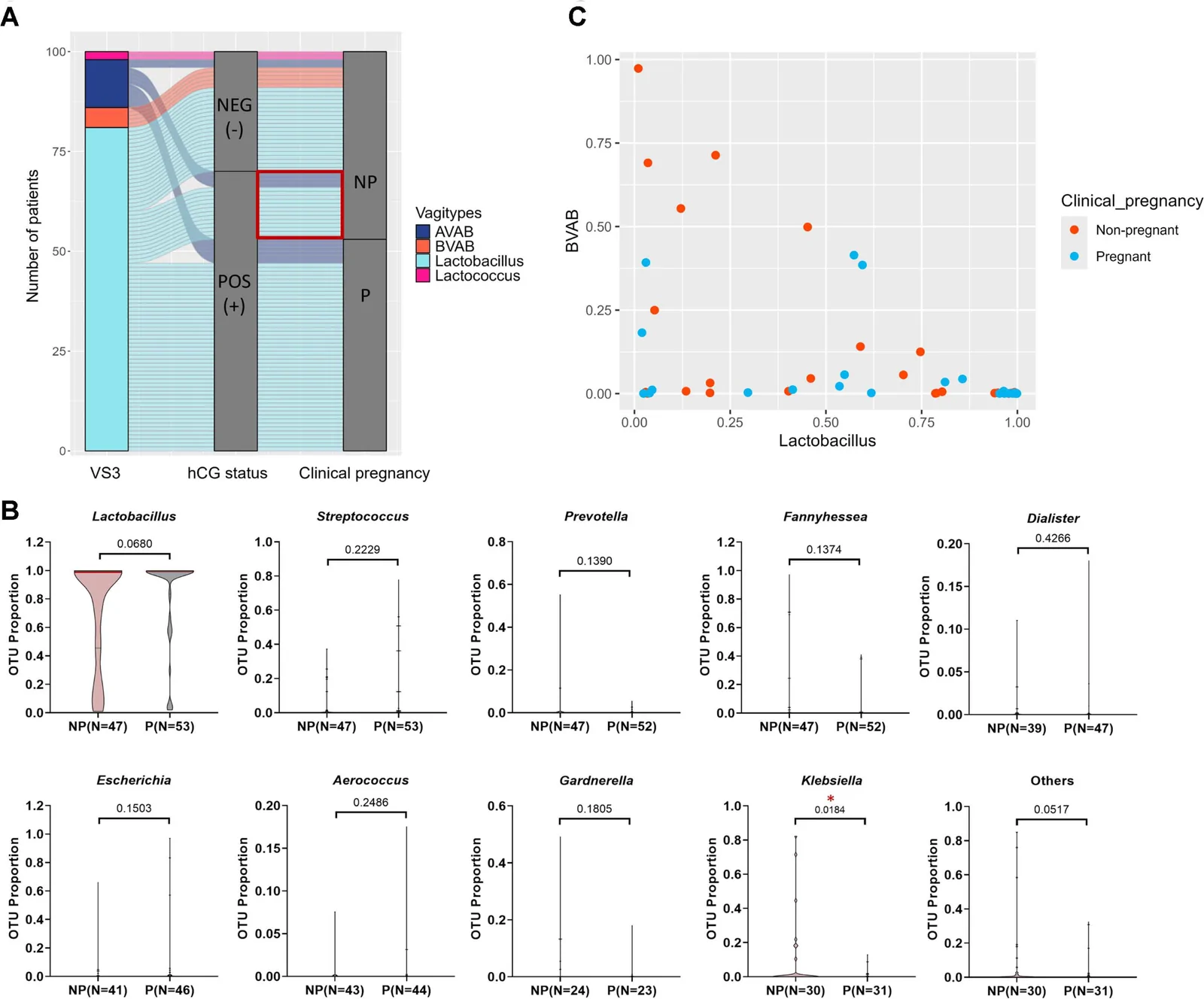
Specific Vaginal Pathogen is associated with early embryonic loss. (**A**) The alluvial plot shows the proportion of each vagitype at the time of the hCG pregnancy test (VS3) and the proportion shift in the subsequent clinical pregnancy examination. (**B**) Violin plots illustrating the relative abundance of various bacterial taxa at the Genus level (*Lactobacillus*, *Streptococcus*, *Prevotella*, *Fannyhessea*, *Dialister*, *Escherichia*, *Aerococcus*, *Gardnerella*, *Klebsiella*, and Others) in pregnant (**P**) and non-pregnant (NP) groups. (**C**) A 2D plot depicting the proportion of the vaginal microbiota vagitype at VS3, differentiated by clinical pregnancy status (pregnant (blue dots) and non-pregnant (red dots). The statistical significance levels: * *P*-value < 0.05, ** *P*-value < 0.01, *** *P*-value < 0.001.

**Fig. 5:**
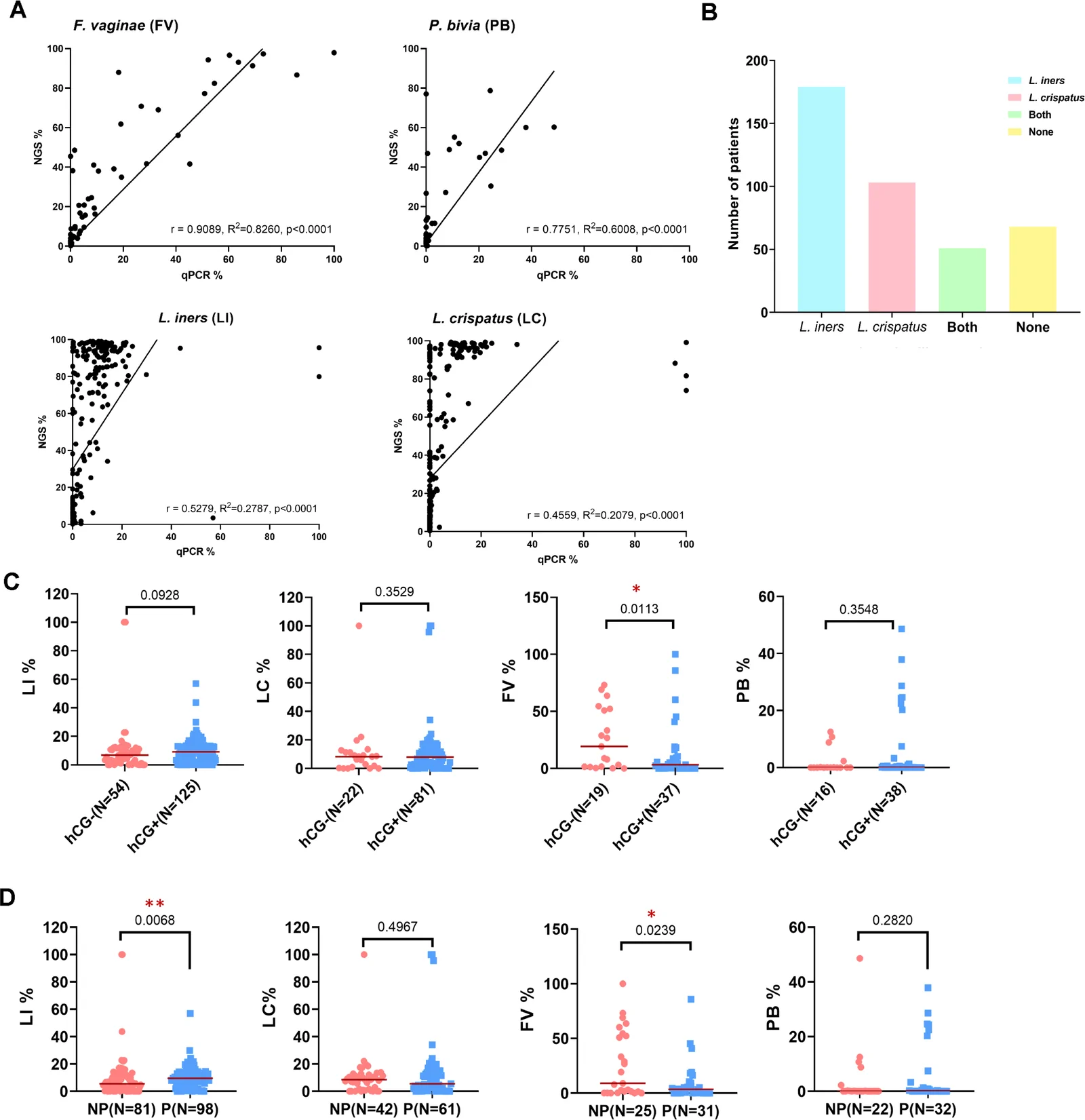
qPCR confirmation of Bacterial Abundance and Pregnancy Outcomes in IVF. (**A**) Scatter plots illustrating the correlation between bacterial abundance measured by next-generation sequencing (NGS) and quantitative PCR (qPCR) for *F. vaginae* (FV), *P. bivia* (PB), *L. iners* (LI), and *L. crispatus* (LC). The correlation coefficient (r), R-squared (R^2^), and p-value are provided for each plot. (**B**) A bar chart demonstrating the distribution of qPCR detection across different bacterial groups (LI, LC, Both, and None). (**C**) Plots comparing the relative abundance (qPCR%) of FV, LI, LC, and PB in hCG-positive and hCG-negative groups. qPCR% represents the relative abundance of each target bacterium, normalized to the universal bacterial 16S rRNA gene (representing total bacteria) using the ΔCt method and expressed as a percentage of total bacteria. (**D**) Plots showing the relative abundance (qPCR%) of FV, LI, LC, and PB in pregnant (**P**) and non-pregnant (NP) groups. The statistical significance levels: * *P*-value < 0.05, ** *P*-value < 0.01, *** *P*-value < 0.001.

**Table 1:** The participant's demographic information by clinical pregnancy outcome.

Demographic information	Participants	Pregnant	Non-pregnant	*P*-value^a^
**Ages**				
**< 35 years**	**41**	**27**	**14**	0.0318^*^
**≥ 35 years**	**59**	**26**	**33**	
**Reproductive condition**				
**With reproductive diseases**	**61**	**29**	**32**	**0.1713**
Endometriosis	5	3	2	
PCO	5	3	2	
Cervical myoma	15	7	8	
Endometrial polyps	32	16	16	
Adenomyosis	4	1	3	
Ovarian cyst	2	1	1	
Oviduct obstruction	19	13	6	
**Without reproductive diseases**	**39**	**24**	**15**	
**History of surgery**				
**Yes**	**37**	**19**	**18**	**0.7917**
**No**	**61**	**33**	**28**	
**Infertility Status**				
**Primary infertility**	**68**	**33**	**35**	**0.1916**
**Secondary infertility**	**32**	**20**	**12**	
**History of infertility treatment**				
**No history**	**50**	**26**	**24**	**0.8412**
**With history**	**50**	**27**	**23**	
**Menstrual cycle**				
**Normal**	**82**	**42**	**40**	**0.5677**
**Abnormal**	**17**	**10**	**7**	
**Hormonal therapy**				
**Hyper**	**15**	**9**	**6**	**0.4578**
**Normal**	**80**	**43**	**37**	
**Poor**	**4**	**1**	**3**	
**History of vaginal infection**				
**Yes**	**27**	**9**	**18**	0.0166^*^
**No**	**73**	**44**	**29**	

^a^The participant's demographic information data were analyzed by using a chi-squared test.

^*^P-value < 0.05.

**Table 2:** List of the identified vagitypes across three IVF stages at the species level.

List of identified vagitypes	Vagitypes distribution among three stages (%)			Patients with vagitypes (N)		
	VS1	VS2	VS3	VS1	VS2	VS3
*Lactobacillus iners*	35	37	28	47	49	37
*Lactobacillus* spp.	25	31	44	24	30	43
*Atopobium vaginae*	39	44	17	7	8	3
*Streptococcus agalactiae*	58	25	17	7	3	2
*Prevotella* spp.	64	27	9	7	3	1
*Streptococcus anginosus*	33	50	17	2	3	1
*Gardnerella* spp.	60	20	20	3	1	1
*Escherichia coli*	20	0	80	1	0	4
*Klebsiella* spp.	0	0	100	0	0	3
*Streptococcus* spp.	0	100	0	0	2	0
*Enterococcaceae*	0	0	100	0	0	1
*Sneathia* spp.	100	0	0	1	0	0
*Lactobacillus reuteri*	0	0	100	0	0	1
*Streptococcus luteciae*	0	0	100	0	0	1
*Lactococcus* spp.	0	0	100	0	0	1
*Lactococcus garvieae*	0	0	100	0	0	1
No vagitypes	50	50	0	1	1	0
