# Supplementary material for: Elevated Level of Fannyhessea vaginae Correlated with Implantation Outcomes in Women undergoing in vitro fertilization Treatment
Source: J Microbiol Biotechnol. 2026 Oct 13;36:e2606022. doi: 10.4014/jmb.2606.06022 (PMC13725140; doi:10.4014/jmb.2606.06022)
Supplement: Supplementary file 1 [file jmb-36-e2606022-supple.pdf]

J. Microbiol. Biotechnol. 2026. 36: e2606022
https://doi.org/10.4014/jmb.2606.06022

Table S1. The participant's demographic information by chemical pregnancy outcome

Demographic information	Participants	Pregnant	Non-pregnant	*P*-value[a]
Ages				
< 35 years	41	33	8	0.0564
≥ 35 years	59	37	22	
Reproductive condition				
With reproductive diseases	61	39	22	0.0978
Endometriosis	5	3	2	
PCO	5	3	2	
Cervical myoma	15	7	8	
Endometrial polyps	32	16	16	
Adenomyosis	4	1	3	
Ovarian cyst	2	1	1	
Oviduct obstruction	19	13	6	
Without reproductive diseases	39	31	8	
History of surgery				
Yes	37	26	11	0.9814
No	61	43	18	
Infertility Status				
Primary infertility	68	43	25	0.0314
Secondary infertility	32	27	5	
History of infertility treatment				
No history	50	35	15	>0.9999
With history	50	35	15	
Menstrual cycle				
Normal	82	55	27	0.2122
Abnormal	17	14	3	
Hormonal therapy				
Hyper	15	13	2	0.0524
Normal	80	56	24	
Poor	4	1	3	
History of vaginal infection				
Yes	27	17	10	0.3504
No	73	53	20	

[a]The participant's demographic information data were analyzed by using a chi-squared test, *p*-value < 0.05

Table S2. List of the Top 20 OTUs Across Three IVF Stages at Species Level.

List of the Top 20 OTUs	Average OTUs (%)			Patients with OTU (N)		
	VS1	VS2	VS3	VS1	VS2	VS3
Lactobacillus iners	44.236	45.948	35.033	100	100	100
Lactobacillus spp.	26.118	31.341	42.749	100	100	100
Fannyhessea vaginae	6.875	7.406	3.953	100	100	100
Streptococcus agalactiae	6.264	3.322	2.471	100	100	100
Prevotella spp.	5.807	2.192	0.880	100	100	99
Streptococcus anginosus	1.610	1.927	0.988	95	97	93
Gardnerella spp.	2.442	1.080	0.911	79	80	88
Escherichia coli	0.692	0.289	3.371	95	95	97
Klebsiella spp.	0.014	0.342	2.992	13	29	73
Streptococcus spp.	0.516	1.456	0.020	96	97	96
Dialister spp.	0.630	0.444	0.408	95	90	99
Enterococcaceae	0.011	0.326	1.050	31	41	73
Aerococcus spp.	0.474	0.334	0.322	100	89	100
Sneathia spp.	1.052	0.021	0.004	86	75	63
Megasphaera spp.	0.193	0.340	0.425	45	64	60
Lactobacillus reuteri	0.053	0.064	0.762	43	55	86
Streptococcus luteciae	0.125	0.094	0.634	21	27	43
Lactococcus spp.	0.000	0.000	0.713	2	1	31
Coriobacteriaceae	0.307	0.148	0.163	76	68	64
Lactococcus garvieae	0.000	0.000	0.559	0	0	26
Others	2.580	2.927	1.595	100	100	100

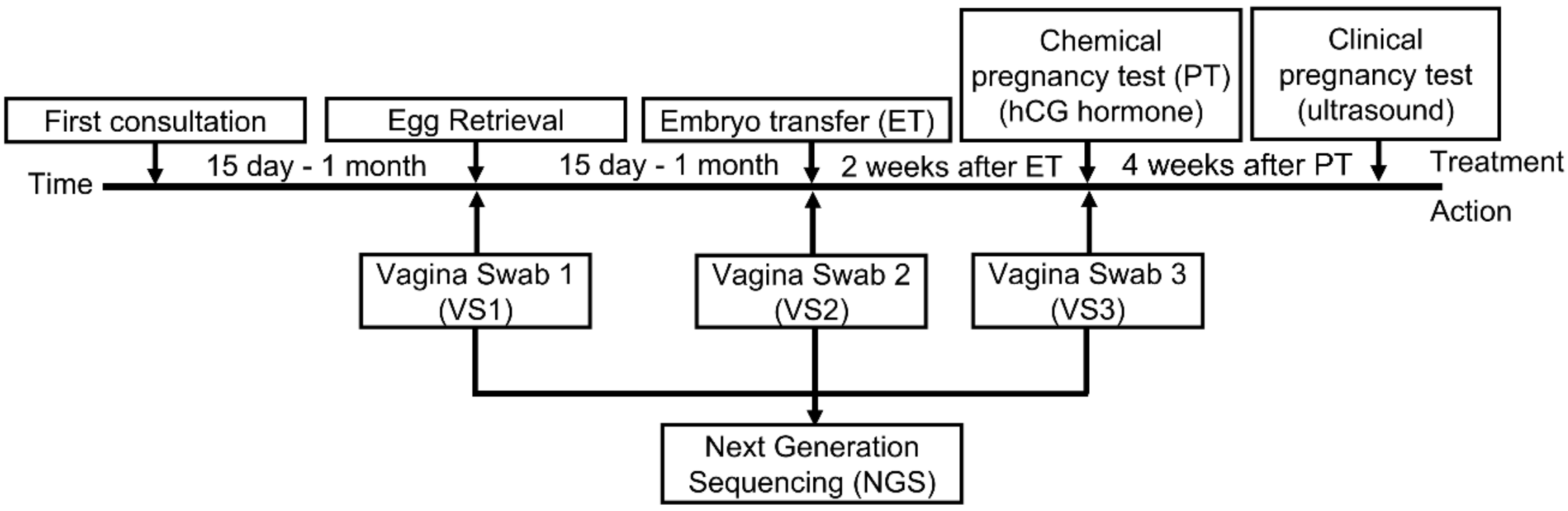

Fig. S1. Schematic diagram of the sample collection plan. Vaginal swabs were obtained at the three phases of IVF treatment during oocyte retrieval, embryo transfer, and hCG pregnancy confirmation. DNA was extracted and subjected to 16S rRNA V3–V5 hypervariable region sequencing using Next Generation Sequencing (NGS).
